# The Antibody Germline/Maturation Hypothesis, Elicitation of Broadly Neutralizing Antibodies Against HIV-1 and Cord Blood IgM Repertoires

**DOI:** 10.3389/fimmu.2014.00398

**Published:** 2014-08-28

**Authors:** Ponraj Prabakaran, Weizao Chen, Dimiter S. Dimitrov

**Affiliations:** ^1^Protein Interactions Group, Laboratory of Experimental Immunology, Cancer and Inflammation Program, Center for Cancer Research, National Cancer Institute, National Institutes of Health, Frederick, MD, USA; ^2^Basic Science Program, Leidos Biomedical Research, Inc., Frederick, MD, USA

**Keywords:** HIV-1, vaccine, broadly neutralizing antibody, cord blood IgM, germline antibody, 454 sequencing

## Abstract

We have previously observed that all known potent broadly neutralizing antibodies (bnAbs) against HIV-1 are highly divergent from their putative germline predecessors in contrast to bnAbs against viruses causing acute infections such as henipaviruses and SARS CoV, which are much less divergent from their germline counterparts. Consequently, we have hypothesized that germline antibodies may not bind to the HIV-1 envelope glycoprotein (Env) because they are so different compared to the highly somatically mutated HIV-1-specific bnAbs. We have further hypothesized that the immunogenicity of highly conserved epitopes on the HIV-1 envelope glycoproteins (Envs) may be reduced or eliminated by their very weak or absent interactions with germline antibodies and immune responses leading to the elicitation of bnAbs may not be initiated and/or sustained. Even if such responses are initiated, the maturation pathways are so extraordinarily complex that prolonged periods of time may be required for elicitation of bnAbs with defined unique sequences. We provided the initial evidence supporting this antibody germline/maturation hypothesis, which prompted a number of studies to design vaccine immunogens that could bind putative germline predecessors of known bnAbs and to explore complex B cell lineages. However, guiding the immune system through the exceptionally complex antibody maturation pathways to elicit known bnAbs remains a major challenge. Here, we discuss studies exploring the antibody germline/maturation hypothesis as related to elicitation of bnAbs against HIV-1 and present our recent data demonstrating the existence of germline-like precursors of VRC01 antibodies in a human cord blood IgM library.

## Introduction

Elicitation of broadly neutralizing antibodies (bnAbs) targeting the HIV-1 envelope glycoproteins (Envs), the key to an effective HIV-1 vaccine, remains elusive. Previous studies have demonstrated several properties of the HIV-1 Envs that could limit their ability to elicit bnAbs. These include protection of the conserved structures by variable loops (1–3), remarkable genetic diversity (4), a glycan shield (5), steric occlusion (6), and conformational masking (7). Until 6 years ago, only a handful of bnAbs, including b12, 2G12, 2F5, and 4E10, were known. Although the structural and functional studies of those bnAbs revealed some important neutralization epitopes (8), such bnAbs have not been successfully elicited by any vaccination approach.

In 2007, we first noted that in HIV-1 specific bnAbs the number of amino acid mutations from their closest corresponding germline sequences was significantly higher than that of bnAbs against the SARS CoV coronavirus, and Hendra and Nipah viruses, which cause self-limiting acute infections (9). Using a large non-immune IgM library, we identified several HIV-1 Env specific antibodies and found that they had fewer somatic mutations than the HIV-1 bnAbs, as well as limited neutralizing activity (9). These findings indicated that elicitation of HIV-1 bnAbs would require far more extensive maturation processes than those needed to generate the bnAbs against the SARS CoV and henipaviruses. So, we have suggested that the difficulty of eliciting these bnAbs may be due, at least in part, to the complex and prolonged maturation pathways required for the development of bnAbs against the HIV-1, which can take long time (10). We thus speculated that this may represent another significant challenge in the development of effective HIV-1 vaccines.

We quantified the number of mutations in human monoclonal antibodies (mAbs) that we selected from phage libraries generated from an HIV-1-infected patient with a known time of infection (10). We calculated the number of amino acid mutations per heavy chain V gene, and defined it as antibody somatic mutational diversification (ASMD). We compared the extent and dynamics of the ASMD between HIV-1-specific mAbs and a panel of SARS CoV-specific mAbs. Our experiments based on the ASMD predicted that elicitation of HIV-1-specific bnAbs would take at least 3 years. An illustrative mathematical model using the ASMD rate based on an exponential time dependent function suggested that a much longer time would be needed for the required maturation, unless somatic diversification had already been initiated from an intermediate antibody. Thus, all these initial studies corroborated our hypothesis that the infrequent occurrence or absence of bnAbs in HIV-1-infected patients could be due, at least in part, to the lack or limited availability of B cell receptors that rapidly mature into bnAbs. Therefore, we suggested that appropriate immunization protocols of long duration need to be developed using the knowledge gained from the exploration of antibody maturation pathways in humans (10).

From the striking observation that all known potent HIV-1 bnAbs are highly divergent from their putative germline predecessors in contrast to bnAbs against henipaviruses and SARS CoV coronavirus, we hypothesized that, since the germline antibodies are so different compared to the highly somatically mutated HIV-1 bnAbs, they may not bind to the Env. This led us to the hypothesis that the immunogenicity of the highly conserved epitopes on the HIV-1 native envelope glycoproteins (Envs) is reduced or eliminated by their very weak or absent interactions with germline antibodies, which could not initiate and/or sustain immune responses leading to elicitation of bnAbs: even if immune responses are initiated and sustained, the maturation pathways are so complex that help and long times may be needed for their elicitation. To test our antibody germline/maturation hypothesis, we designed germline-like antibodies corresponding to the known bnAbs b12, 2G12, 2F5, X5, m44, and m46 (the latter three antibodies were discovered in our laboratory and possess HIV-1 cross-reactivity with moderate neutralizing activities) and evaluated them for binding to Envs (11). We found that while germline-like X5, m44, and m46 bound to Envs with relatively high affinity, the germline-like precursors of b12, 2G12, and 2F5 failed to bind Envs in an ELISA assay although their corresponding mature bnAbs bound strongly. These results provided initial evidence that the Env structures containing conserved epitopes might not initiate humoral responses due to limited or absent binding to the germline precursors of bnAbs. These germline precursors may also be of limited availability as recently reported (12).

Following that initial study, we expanded our investigation to different variants of the two different antibodies (b12 and X5) including their closest germline counterparts and several germline-like intermediates (13). The experiments showed that b12 intermediate antibodies neutralized only some HIV-1 isolates with relatively weak potency. In contrast, intermediates of X5 neutralized a subset of the tested HIV-1 isolates with efficiencies comparable to those of the matured X5. These results helped explain the relatively high immunogenicity of the coreceptor binding site on gp120 and the abundance of CD4-induced (CD4i) antibodies in HIV-1-infected patients (X5 is a CD4i antibody) as well as the maturation pathway of X5. In the case of b12, germline-like intermediates along the maturation pathway were shown to not only bind some Envs but also human self antigens, suggesting that antigens other than the Envs could help guide the immune system through the b12 maturation pathway.

Therefore, we proposed a conceptually new vaccination approach, in which it is critical to identify primary immunogens that bind to the germline antibodies that are predecessors of bnAbs. If needed, these immunogens should be combined with secondary immunogens that recognize intermediate and/or matured antibodies to guide the immune system through the prolonged, complex maturation pathways (14). In this respect, we envisioned that the knowledge of human antibodyomes would become indispensable to elucidate the origin, diversity, and maturation pathways of bnAbs and discover germline-like intermediates of bnAbs that could provide a basis for the design of novel HIV-1 vaccine immunogens (14, 15).

In recent years, several groups have reported a number of new bnAbs that were identified from multiple HIV-1 infected individuals using designed novel antigen baits and advanced technologies implemented in isolating human mAbs and high-throughput sequencing (16). Particularly, Haynes, Kwong, Stamatatos, Scheif et al. have dealt with a large amount of data delineating structural, genetic determinants, and maturation pathways of different bnAbs. These studies not only confirmed our previous findings that the Envs fail to engage germline versions of bnAbs but also suggested possible holes in B cell repertoires and demonstrated the implications of our antibody germline/maturation hypothesis for finding germline-like precursors, intermediates as well as for designing immunogens that could potentially bind to such bnAb intermediates. In this report, we discuss the recent advancements in HIV-1 vaccine research in the context of the antibody germline/maturation hypothesis, and highlight critical factors to be considered when exploring germline-like precursors and intermediates of bnAbs. We also report for the first time using 454 sequencing data analysis of a human cord blood IgM library to identify putative germline precursors of the heavy and light chains of VRC01-like antibodies. These naturally occurring cord blood-derived VRC01-like heavy and light chains may be useful as putative templates for designing novel vaccine immunogens that can lead to the elicitation of VRC01-like antibodies and for understanding the maturation pathways of this bnAb. Still there are major challenges to be overcome. New empirical and semi-empirical approaches could be successful; recently, new paradigms were discussed that could better fit our increased knowledge of HIV immunopathology and which could possibly be more helpful in guiding future vaccine research than did past unsuccessful approaches (17).

## Materials and Methods

### Antibodyome database and tools

DNA isolation, amplification, and 454 sequencing of the human cord blood IgM library were previously described in detail (18, 19). For quality control, we trimmed the 454 sequence reads and retained only sequences with lengths of more than 300 nucleotides, covering the entire antibody variable domains consisting of all three complementarity determining regions (CDRs) along with framework regions (FRs). We used IMGT/HighV-QUEST for immunogenetic and statistical analyses (20). The output results from the IMGT/HighV-QUEST analysis were stored in a local PostgreSQL database, and structured query language (SQL) was used to retrieve the data for further analysis. Statistical calculations were carried out using JMP10^®^ statistical software (SAS Institute, Cary, NC, USA).

### Computational analysis of antibody sequences

Antibody sequences from IGHV1-2 and IGK3-11 lineages were retrieved from our local antibodyome database consisting of immunogenetic data derived from 454 sequencing of the human cord blood IgM library using SQL statements. Amino acid sequence identities between each of the selected lineage sequences from the 454 sequence data and pertinent germline sequences were calculated based on the pairwise alignment using local BLAST as implemented in BioEdit v7.0.9 (21). Phylogenetic analysis was carried out using the Archaeopteryx software (22).

## Results and Discussion

### Exploring the antibody germline/maturation hypothesis

**Figure 1:**
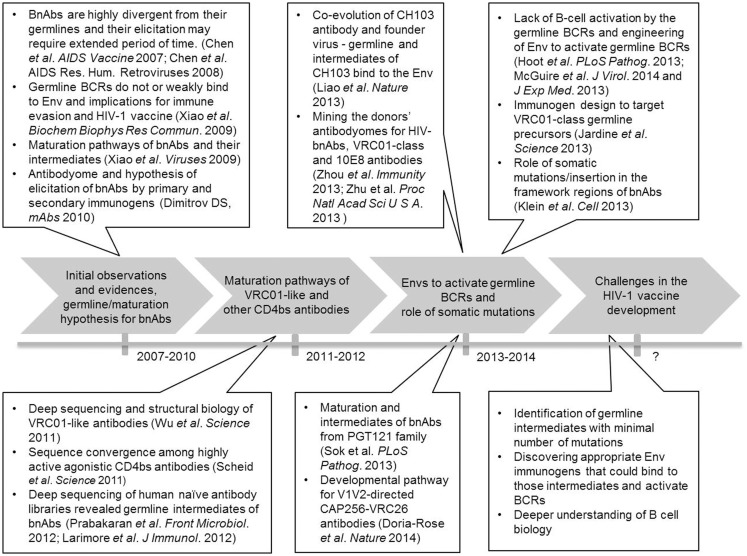
**Timeline of the antibody germline/maturation hypothesis and experiments**.

Our earlier observation of the extensive maturation of HIV-1 bnAbs in contrast to those against some viruses causing acute infections led to the antibody germline/maturation hypothesis (9–11, 13, 14). According to this hypothesis, it is critical to identify immunogens that would bind to germline and/or intermediate antibodies of bnAbs, as well as the exploration of antibodyomes could be useful for identifying such immunogens (14). Figure 1 describes the timeline involving some of the key developments in current HIV-1 vaccine research focused on antibody germline-like intermediates and maturation pathways of bnAbs. Major research efforts in this direction were spearheaded by deep sequencing and structural biology studies of VRC01-like and other CD4-binding site (CD4b) antibodies from HIV-1-infected individuals. These studies delineated possible maturation pathways of such antibodies with high levels of somatic mutations and convergence in antibody recognition (23, 24). Both studies revealed that the putative germline precursors of these antibodies had weak or no apparent affinity for Env, and acquisition of a large number of somatic mutations were needed for the breadth and potency of these antibodies. These studies also explored antibody diversity and found many intermediates of similar lineages of the heavy chain genes from the two IGHV families VH1-2 and VH1-46 that paired with different light chain genes. Thus, analysis of the VRC01-related antibodyome from HIV-1 infected patients revealed B cell maturation pathways that may help guide the vaccine-induced elicitation of such antibodies. However, if we could find germline-like intermediates of such bnAbs from a naïve antibody repertoire, then potential vaccine immunogens developed based on those templates would stimulate an adequate B cell immune response in healthy humans. To this end, we identified VRC01-like intermediate antibodies from a naïve antibody library of human cord blood, which is presented later in the text.

We previously analyzed the IgM repertoires of healthy individuals and identified several intermediates of b12 from the VH1-3 gene family (15). Sequence analysis of 28,925 unique sequences from the IgM repertoires revealed a CDRH3 with a length (20 amino acids) and sequence similar (50%) to that of the b12 CDRH3, but the V gene associated with that CDRH3 was found to be HV4-b (15). This finding indicates that long CDRH3s may not be a limiting factor for the development of bnAbs (25) although long CDRH3 motifs with certain amino acid preferences and/or associations with particular heavy or light chain families favoring polyreactivity may not be undermined.

Stamatatos and coworkers have conducted experiments screening a large panel of recombinant Envs for binding to the germline predecessors of b12, NIH45-46, and 3BNC60 to test how Env immunogens interact with the predicted germline versions of known bnAbs (26). They found that the mature bnAbs reacted with diverse Envs but the corresponding germline antibodies did not. They examined in detail the germline b12 and its chimeric forms – either the germline heavy chain paired with the mature light chain and vice versa – to test whether they could interact with any of the recombinant Envs derived from clade A, B, and C viruses. Among all the recombinant Envs tested, at least one Env (QH0692) was found to bind a b12 chimera with a mature heavy chain. However, this chimera failed to mediate calcium mobilization, indicating no BCR activation via BCR-antigen engagement. In other studies, they found that the elimination of certain conserved glycosylation sites on Envs led to the binding of germline versions of VRC01 and NIH45-46 and BCR activation (27) but that the modified Envs did not interact with PG9 and 447-52D germlines (28).

Haynes and coworkers have succeeded in finding Envs capable of engaging the germline versions of a CD4bs bnAb, CH103, while studying the co-evolution of the antibody in an HIV-1 infected patient (29). They found that CH103 is less mutated than most other CD4bs bnAbs, and importantly that the unmutated common ancestor of the CH103 lineage avidly bound the transmitted/founder HIV-1 Envs. This finding suggests that early founder Envs could bind optimally to the germline and intermediate versions of CH103, and therefore, are promising vaccine immunogens, representing an important step forward in HIV-1 vaccine development.

Similarly, the maturation pathway of the potent V1V2-directed HIV-neutralizing antibody, CAP256–VRC26, has been described, in which a germline-like intermediate with a 35-amino acid residue long CDRH3 was shown to bind and neutralize the superinfecting virus weakly, but did not bind or neutralize heterologous viruses (30). These results suggest that the CAP256–VRC26 lineage could be initiated by using a rare superinfecting-virus-like V1V2 Env.

In another successful effort in identifying an Env that could engage the germline versions of bnAbs, Scheif and coworkers devised a computation-guided approach combined with *in vitro* screening to engineer a gp120 outer domain. The designed protein not only bound to multiple VRC01-class bnAbs and their germline precursors but also activated B cells expressing diverse intermediates of the bnAbs (31). Therefore, priming with the protein and subsequent boosting with more native immunogens could help induce early somatic mutations and the ultimate elicitation of VRC01-class bnAbs.

Interestingly, Nussenzweig and coworkers’ study showed that somatic mutations of the FRs and insertions of some bnAbs are required for their broad and potent HIV-1 neutralizing activity (32). Based on structural information, they made different germline versions of VRC01, NIH45–46, 12A21, and 3BNC117, and found that mutations in FRs were also essential for binding, breadth, and potency of most bnAbs. This suggested that certain framework mutations could be critical and should be preserved for designing the intermediates of such bnAbs. Several other studies mining the HIV-1 infected donors’ antibodyomes (33–35) revealed putative intermediates of bnAbs. Many of them with lower levels of somatic hyper mutations could bind to selective Envs; for example, intermediates of PGT121-134 were able to preferentially bind native Envs relative to monomeric gp120 (36). We also identified 2F5-like antibodies (m66 and m66.6) with much fewer mutations than 2F5 and suggested their use as a model system for elicitation of such antibodies (37, 38).

All these newly discovered bnAbs raise the hopes for effective HIV-1 vaccine development as they reveal characteristic features of bnAbs that could help us understand the immunological basis critical for their production and also serve as templates for rational vaccine design. Therefore, the focus has been dramatically shifted to explore and overcome the immunological hurdles associated with the elicitation of bnAbs, namely, extensive somatic mutations of bnAbs. Major challenges remain in identification of intermediates with a minimal number of mutations, and appropriate Env immunogens that would bind such intermediates and activate BCRs, which can lead to the maturation of the intermediate antibodies to bnAbs. Recently, new paradigms that better fit our increased knowledge of HIV immunopathology and which may be more helpful in guiding future vaccine research than did past unsuccessful approaches were discussed (17).

### Identification of putative germline-like intermediates in the maturation pathways of VRC01

**Figure 2:**
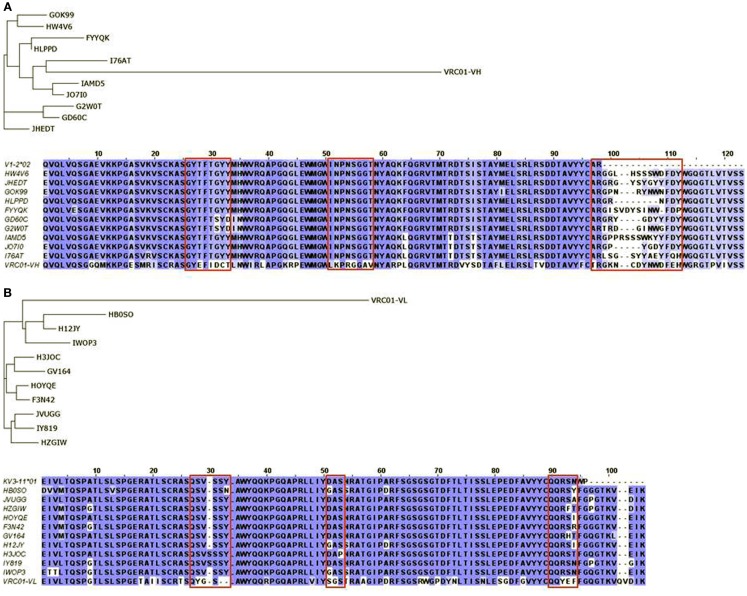
**Phylogram and sequence alignment of selected (A) heavy and (B) light chain sequences found in human cord blood IgM libraries of IGHV1-2 and IGKV3-11 lineages, respectively**. Phylogenetic trees constructed using the amino acid sequences of top 10 putative germline-like precursors as found in the cord blood library along with mature heavy and light chains of VRC01 are shown. Multiple sequence alignments of heavy and light chain sequences along with their corresponding germline sequences, V1-2*02 and KV3-11*01, made using BLOSUM62 scoring matrices, conserved substitution (blue) and non-conserved substitution (white) are shown.

We previously characterized the human cord blood cell-derived IgM antibodies using 454 sequencing to study gene diversity and somatic mutations (19). Naïve germline antibody repertoires, particularly from babies, may be quite unique for understanding the B cell maturation pathways, as they can also mount an immune response against HIV-1 as recently found (39). Our earlier gene usage analysis of the cord blood IgM repertoire showed the biased IGHV gene usages (19) as similar to adult IgM repertoires (40). However, we already noted that the IGHV1-2 gene usage was significantly higher in the cord blood IgM repertoire, i.e., an overall contribution of 20% as compared to 8% in adult IgM repertoires. This suggested that the cord blood IgM repertoire may be advantageous for the exploration of the IGHV1-2*02 lineages when studying germline precursors and intermediates of VRC01 heavy chain. A total of 5,624 heavy chain and 1,096 light chain sequences of IGHV1-2 and IGKV3-11 lineages, respectively, were used to select the top 10 sequences as closest intermediates for VRC01 in each heavy and light chain categories by using local BLAST searching. We performed phylogenetic analysis of the selected sequences to identify genetic relationships among VRC01-like intermediates of heavy (Figure 2A) and light (Figure 2B) chains. We found two of the antibody heavy chains, HWAV6 and JHEDT, which were 100% identical to the IGHV1-2*02 germline sequence. Remarkably, their CDRH3 sequences had the same length (14 amino acids) as that of the VRC01 heavy chain. For these 10 heavy chain sequences, the CDRH3 lengths ranged from 8 to 16 amino acids with sequence variations at the junctions. One of the germline sequences, JHEDT, had a point mutation at Cys100Tyr (Kabat numbering) of CDRH3 that exactly mimicked the residue Tyr100 of CDRH3 in VRC01. The residue Tyr100 at CDRH3 of VRC01 is most likely contributed by the IGHD3-16*02 germline with a point mutation Cys100Tyr. The other heavy chain sequence I76AT, which was the closest to VRC01 heavy chain, also had the same mutation at Cys100Tyr. One of the other germline sequences, HWAV6, had Trp100B (Kabat numbering) of CDRH3 that exactly mimicked the residue Trp100B of CDRH3 in VRC01. Intriguingly, the Trp100B residue is a junctional amino acid of the CDRH3 in germline HWAV6, and it exactly replicates the Trp100B junctional residue of CDRH3 in VRC01. This suggests a possible maturation mechanism involved in the VRC01-like intermediates where junctional amino acids could determine the maturation pathway far preceding the somatic hypermutation required for affinity maturation (41). Most of the closest IGHV genes, 8 out of 10 shown in the Figure 2A, have at least one mutation in the V region, and two sequences, G2W0T and GD60C, have two mutations at each of the CDRH1. The pre-existing amino acid mutations found in the V region and CDRH3 sequence information may inform the design of heavy chain germline-like precursors and intermediates, and help naturally reconstruct the B cell clonal lineages in the maturation pathways of VRC01.

Light chain recognition of Envs by VRC01 and VRC01-related antibodies has been studied in detail using structural and 454 sequencing data (33). The VRC01 light chain commonly uses the IGKV1-33 lineage and has a characteristic five amino acid long CDRL3 and a distinctive two amino acid deletion in CDR L1. Therefore, we selected the IGKV1-33 lineage sequences with five amino acid length CDRL3s, but no sequences were found with a two amino acid deletion in CDR L1 (Figure 2B). All of them had either framework or CDR mutations or both. Four of them had a point mutation at CDRL1 and seven of them had a point mutation at CDRL3.

The structural basis for germline gene usage of VRC01-related antibodies targeting the CD4bs has been previously described (42), which revealed a set of signature features for these antibodies that were verified by mutagenesis. These signature features explained the origin of the IGVH1-2 gene and antibody resistance for some Env sequences. We found that characteristic residues including the Trp100B of heavy chains were conserved while light chains did not have any characteristic residues as reported previously (42). However, other pre-existing amino acid mutations in light chains could have implications for the VRC01-related intermediates with a characteristic CDRL3 of five amino acid length.

### Distributions of CDR lengths and amino acids in the VRC01-related germline genes

**Figure 3:**
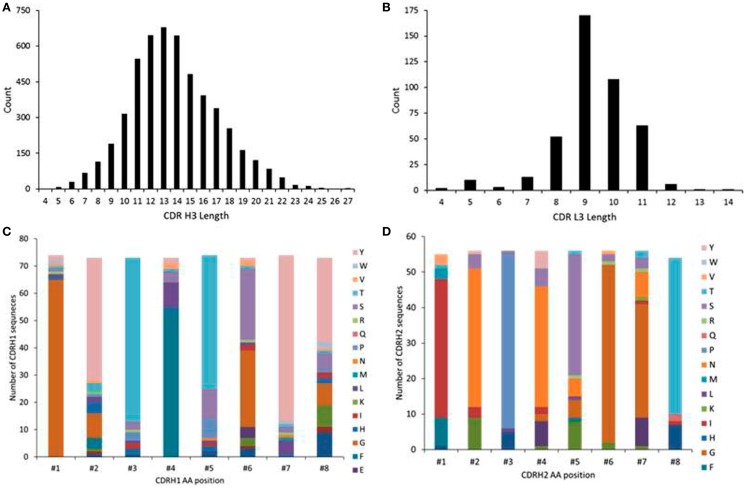
**Distributions of amino acid length in (A) CDRH3, (B) CDRL3 and amino acid compositions in (C) CDRH1 and (D) CDRH2 as derived using the gene families of IGHV1-2 and IGKV3-11 in human cord blood IgM library showing the CDR3 lengths diversity and pre-existing mutations at CDRH1 and H2**.

We analyzed the amino acid length distributions of CDRH3 and CDRL3 sequences that were of VRC01 origins, namely, IGHV1-2 and IGKV3-11 for heavy and light chains, respectively, as derived from the human cord blood IgM library (Figure 3). The CDRH3 lengths ranged from 4 to 27 amino acids, indicating high CDR3 length diversity (Figure 3A). VRC01 has a CDRH3 length of 14 amino acids, which is shorter than those of most other anti-HIV-1 antibodies (25). The LCDR3 lengths ranged from 4 to 14 amino acids (Figure 3B). The CDRL3 of VRC01 has a characteristic length of five amino acids with a mature genetic signature (33). Analysis of the human cord blood IgM repertoire showed only a fraction of such light chains with a shorter length of five amino acids (Figure 3B). Frequency distributions of amino acid compositions of CDRH1 and CDRH2 in IGHV1-2 sequences as observed in the human cord blood IgM repertoire are plotted in Figures 3C,D, respectively. These plots show that there are position specific variations in the CDRH1 and CDRH2 regions of IGHV1-2 genes. These could indicate possible IGHV1-2 specific pre-existing amino acid mutations in CDRH1 and CDRH2, as observed in several naïve antibody heavy chain sequences, which could inform the design of germline precursors and intermediates of VRC01-like antibodies.

### V–D–J recombination diversity and IGHD reading frame usages in the VRC01-related germline genes

**Figure 4:**
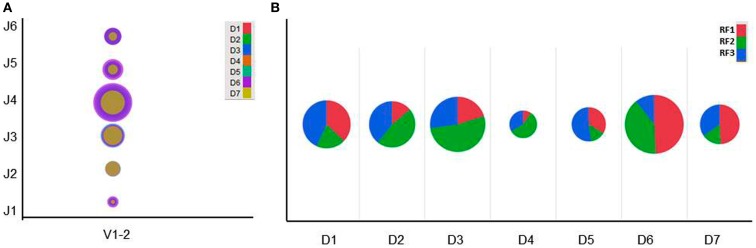
**(A)** Bubble plot showing the V–D–J diversity associated with IGHV1-2 gene sequences and **(B)** Pie chart showing the IGHD reading frame (RF) usages in human cord blood IgM library.

We previously observed that the V–D–J rearrangement patterns occurred at different frequencies with 1,430 V–D–J combinations in a human cord blood IgM repertoire (19). Figure 4A shows the V–D–J diversity associated with IGHV1-2 gene sequences using a bubble plot for comparison with different D and J genes. The VRC01 heavy chain uses IGHD3-16 and IGHJ2 genes to recombine with IGHV1-2. However, other VRC01-related antibodies exhibit a skewed usage of IGHJ genes although at least three different IGHJ genes (IGHJ1, IGHJ2, and IGHJ4) are involved (23). As the human cord blood IgM library has a large functional V–D–J diversity, it can be used to identify potential VRC01-like heavy chain germline precursors and intermediates.

In jawed vertebrates the expressed heavy chains may use any of the six IGHD reading frames (RFs); however, RF1 is thought to be the preferred one as it mostly encodes tyrosine and glycine. The remaining five RFs encode either hydrophobic or charged amino acids, but the use of inverted RF1, RF2, and RF3 are discouraged. Preferential usage of IGHD RFs has been long implicated in B cell development and antigen-specific antibody production (43–45), and selected based upon its amino acid content (46). Genetic control of IGDH RF preference over the regulation of repertoire development has been recognized (47). Here, we have analyzed the productive IGHD RF usages in a human cord blood IgM library. Frequency distribution of RFs is plotted using a pie chart as depicted in Figure 4B. We noted that there were not any highly restricted usages of the IGHD RFs although some preferential usages depending on the IGHD genes were found. This clearly indicates that IGHD RFs diversity could add more diverse amino acid contents leading to enormous CDRH3 diversity. It may also be possible that intermediates with different RF choices play a critical role in selecting certain maturation pathways efficiently.

## Conclusion

The antibody germline/maturation hypothesis led to a paradigm shift in the design of immunogens for bnAb elicitation, as well as the realization of the importance of the complexity of the bnAb maturation pathways, and exploration of human antibodyomes (14). In fact, human antibodyome exploration is also promising for other fields of science and medicine (14, 48). This antibodyome approach is now a major direction of research in the HIV-1 vaccine field (16, 49). An important goal is to precisely identify naturally occurring germline-like precursors and intermediates of bnAbs that could help designing novel immunogens, which could activate the corresponding BCRs and drive the immune system to produce bnAbs within a short period of time. We presented an approach using a human cord blood IgM library to identify putative germline precursors and intermediates of VRC01-like heavy and light chains, which could be useful in reconstructing the B cell clonal lineages in the maturation pathways of VRC01-related bnAbs. This method has the potential to help in the identification of naturally occurring germline-like precursors and intermediates of any known bnAb and in the development immunogens based on HIV-1 Envs (50) and peptides (51), as well as non-HIV-1 molecules (12). However, major challenges remain and new paradigms that better fit our increased knowledge of HIV immunopathology could possibly be more helpful in guiding future vaccine research than did past unsuccessful approaches (17).

## Conflict of Interest Statement

The Guest Associate Editor Marc H. V. Van Regenmortel declares that, despite having collaborated with the author Dimiter S. Dimitrov, the review process was handled objectively and no conflict of interest exists. The authors declare that the research was conducted in the absence of any commercial or financial relationships that could be construed as a potential conflict of interest.
